# Health Care Reform Hinges on Private-Sector Collaboration

**Published:** 2009-03-15

**Authors:** Bill Novelli

**Affiliations:** AARP, Office of the CEO

## Abstract

America's health care system is characterized by rising costs, increasing numbers of Americans who lack health insurance coverage, and poor quality of health care delivery. The convergence of these factors is adversely affecting not only the health of Americans but also the ability of businesses to compete successfully in a global marketplace. AARP and other nonprofit organizations are collaborating with the private sector to have more people covered by health insurance and to educate them to make behavioral choices that prevent chronic disease and ultimately lower costs.

## Introduction

America's health care system is broken. It is characterized by the convergence of 3 dangerous trends: rising costs, increasing numbers of Americans who lack health insurance coverage, and poor quality of health care delivery. And it is getting worse. The Center for Studying Health System Change reports that 20% of the population, about 59 million people, delayed or went without necessary medical care in 2007. That's an increase of almost 65% since 2003 (1).

As the chief executive of AARP, a nonprofit, nonpartisan membership organization that represents the needs and interests of people aged 50 and older, my work has shown that health care reform will require the public and private sectors to work together. Neither government nor industry can do it alone. Nor can the problem be solved by attacking only one aspect. A range of strategies is needed to create fundamental reform.

In my book, *Fifty Plus: Give Meaning and Purpose to the Best Time of Your Life* (2), I identify 7 strategies for transforming the system: improve the use of health information technology, reduce medical errors, focus more on treating and managing chronic diseases, lower the cost of prescription drugs, make sure that all Americans have access to health care, promote health and healthy behaviors, and focus more on preventing disease, not just curing it.

This article focuses AARP's work in these last 2 areas — health promotion and disease prevention — in collaboration with for-profit organizations.

## Prevention

AARP's position is that health promotion and disease prevention can change America. Some segments of the health care industry (eg, the Centers for Disease Control and Prevention, the Partnership for Prevention, and the US Preventive Services Task Force). are doing great work to promote health and prevent disease. But many sectors of the health care industry have not made this approach a priority. Our health care system gives us simultaneously the world's preeminent medical research enterprise and more than 47 million people who have no health insurance coverage. This system produces much more voluminous information about specific diseases than it does about how to maintain good health. Our remarkable publicly funded medical research is focused on understanding disease rather than understanding health. Privately funded pharmaceutical research and development is based on creating profitable interventions for disease and disorders. In short, this nonsystem is often more willing to pay for intervention than prevention. A redirected focus in these areas can improve the quality of life for millions of people and make health care affordable for government, corporations, employees, and families as the population ages and becomes more diverse.

Prevention and wellness should be a top national priority. We need to work together to transform our "sick care" system to a "health care" system that values and invests in prevention and wellness. We must move from a system that focuses on treating illness to one that also prevents disease.

The National Business Group on Health found that almost 47% of deaths in the United States in 2000 were caused by modifiable health behaviors; tobacco use, poor diet, and physical inactivity were the largest culprits (3). The Commonwealth Fund estimates that, with a modest investment, we could save the health care system $493 billion over 10 years by reducing the prevalence of tobacco use and obesity, creating incentives for participation in wellness programs and adoption of healthy behaviors, and covering preventive services (4).

Attempts to move toward a health system model that focuses more on health promotion and disease prevention have been successful only to a limited degree. Coherence and synergy are needed to unify the efforts of state and local government agencies, whose programs depend on government funding and political will. Even some successful programs — such as the National High Blood Pressure Education Program at the National Heart, Lung, and Blood Institute or the youth tobacco control programs in Florida and Mississippi — have been severely cut back due to budget constraints or shifts in priorities (2).

This is a complex problem that requires a multifaceted approach. To succeed at health promotion and disease prevention we need to 1) create environmental change, so that appropriate behavior is seen as normative behavior, which means changing the environment in which people actually live, work, and play, and 2) focus on behaviors by teaching people how to take the proper steps to good health or healthy living.

Clinical settings can contribute to both environmental change and individual behavior change. They are an important part of the solution, but people do not live in clinical settings. They live in supermarkets, convenience stores, schools, playgrounds, restaurants, offices and factories. They also live in a media society — in front of televisions, video games, movies, computer screens, and cell phones … and especially on couches.

A major public-private partnership that has broad representation from all stakeholders — including health care, insurance, the food industry, agriculture, educators, consumers, media, advertising, government, and research — is essential to draw attention to the importance of health promotion and disease prevention. Such a broad partnership would make prevention a national priority and provide a focal point for action. It would advance policies and practices to prevent disease and improve health and it would offer national, coordinated leadership for wellness and prevention activities.

## Partnering With Private-Sector Organizations

At AARP, we are reaching out to collaborate with other organizations that share our concerns and goals. Many of these are private, for-profit companies. These companies must be part of the solution, and increasingly, they want to be.

The private sector continues to be hit hard by the high cost of health care, which limits its ability to compete in the global marketplace. These companies seek partners and join coalitions where they can have a greater impact and involve a broader constituency to effect social change.

More and more corporate chief executive officers (CEOs) realize that running a corporation is no longer a matter of maximizing shareholder returns. It is much more a public endeavor, involving many parties and requiring well-honed political skills. Consequently, CEOs must become more like those of us in the public and social sectors; their need to deal with multiple constituencies, to engage and empower stakeholders, and to become "a force for doing good," as Tom Robertson, Dean of the Wharton Business School at the University of Pennsylvania likes to say, has led to a resurgence in corporate social responsibility.

Corporate social responsibility helps companies to motivate, attract, and retain staff, and enhances corporate reputation among customers, which in turn can positively affect the bottom line (5).

Indra Nooyi, CEO of PepsiCo, challenged herself and other CEOs by calling corporations "engines of efficiency" with a mandate to act responsibly and start changing immediately. In a speech to the food industry in January 2008, she pushed the group to tackle obesity. "Let's be a good industry that does 100% of what it possibly can — not grudgingly, but willingly," she said (6).

*The Economist* (5) reports that corporate social responsibility, real or perceived, is rising sharply in global executives' priorities. Yet even as more corporate leaders tout their efforts at corporate social responsibility, few companies are doing it well.

By reaching out to private-sector companies that are serious about corporate social responsibility, AARP can help them, they can help AARP, and together we can bring about significant change and make life better for our 40 million members and their families. The examples below illustrate how we collaborate with private-sector, for-profit partners to bring about change in health promotion and disease prevention.

## Advocacy

To advance its efforts to work with the private sector, AARP started a national grassroots movement — Divided We Fail — with Business Roundtable, which represents the CEOs of America's largest corporations; the Service Employees International Union, the fastest-growing labor union in the country; and the National Federation of Independent Business, which represents America's small businesses (2). The movement is supported by nearly 100 other organizations that share our commitment. Our organizations do not always agree, but we realize we are facing common problems in health care and financial security. We recognize that only by coming together to address these common problems can we achieve our individual goals.

Divided We Fail promotes access to quality, affordable health care (including prescription drugs) for all Americans. Its idea is that wellness and prevention, including the personal responsibility of improving behavior with diet and exercise, should be national priorities. Over the past 2 years, Divided We Fail has worked to engage Congress, other organizations and businesses, AARP members, the public, and the media in the urgent need for health reform. We have advertised extensively, hosted national issue forums and briefings on Capitol Hill, and endorsed legislative solutions on wellness and prevention, health information technology, and transparency of health data.

Divided We Fail supports measures such as the Medicare Quality Enhancement Act, which will help improve health care quality and efficiency and help bring patients the performance data they need to make educated decisions about their care; the Wired for Health Care Quality Act, which would spur adoption of a nationwide health information technology system; and the reauthorization of the State Children's Health Insurance Program.

## Information and Education

In partnership with Walgreens, AARP is working on several projects to create environmental change and help people take the proper steps to good health. As part of our campaign, Wise Use of Medications, we are educating consumers in more than 6,500 Walgreens pharmacies. The campaign educates Walgreens customers how to buy and use prescription drugs, so they can maximize benefits and save money.

Many of our state offices are working with Walgreens and other pharmacies on what we call "brown bag medication reviews." Consumers bring all of their medications, supplements, and vitamins to a community center, where pharmacists consult with them individually about drug compliance and interactions. Five states have piloted AARP "Let's Talk About Meds" seminars to help people manage their medications and enjoy lower costs by using generics.

In April 2009, we plan to launch the AARP/ Walgreens Wellness Tour to deliver free health screenings and health education to people in hundreds of communities, particularly in diverse and underserved areas. Nine buses will make more than 2,000 stops in 300 cities across 48 states and Puerto Rico. The tour will be staffed by trained medical personnel who will provide 1.3 million free screenings for cholesterol, blood pressure, bone density, glucose levels, waist circumference, and body mass index.

After receiving the instant results, visitors to the Wellness Tour will be encouraged to visit their doctor or other health care provider if they have any concerns. They will also receive educational materials to help empower them to take charge of their health.

Both AARP and Walgreens are focused on improving health and quality of life for all Americans aged 50 or older, their families, and others in their communities. We realize that many diseases can be detected long before their symptoms are even noticed and that early detection is the key to staying healthy. By joining forces, we are using our combined influence and broad reach to get relevant and important information to people who need it most.

## Programs and Services

More than advocacy and education are needed to transform the health and well-being of Americans and our health care system. Innovations in the health care marketplace are necessary to cover more people, change behaviors, refocus delivery, and improve financing.

In April 2007, AARP announced an expanded relationship with UnitedHealth Group and a new provider relationship with Aetna to offer health insurance for the members of AARP. We recognize that the current health care delivery system is broken and that the Medicare program is in financial crisis. We understand that the fragmented nature of US health care contributes to rising costs. AARP and UnitedHealth Group are providing leadership in attempting to change the marketplace by improving the efficiency of the health care system with more coordination and better care management.

As many as 22% of Medicare beneficiaries have a supplemental health plan through UnitedHealth Group, but for the first time, AARP Medicare Supplement Plan members will have access to clinical programs and health tools that can guide them to better health. Dedicated case managers will oversee members' care and direct them to resources and tools that will improve their understanding of their health and empower them to live healthier lives.

Separate initiatives will serve all AARP members, nonmembers, and physicians to help with health education, life planning, and coaching.

At the heart of both these agreements with UnitedHealth Group and Aetna are incentives to

- Increase coverage for the uninsured.
- Increase access to and reduce disparities in health care, particularly for underserved populations.
- Provide care management, disease management, geriatric screenings, and depression management programs.
- Provide programs to monitor quality of care and patient satisfaction.

We assess the performance of the plans by measuring quality, efficiency, and consumer experience, and we publicly report results.

In December 2008, UnitedHealth Group and AARP launched a series of Health Care Transformation pilot programs to improve health outcomes for diabetes, heart disease, depression, and other chronic health conditions. AARP Medicare Supplement plan members living in central North Carolina; Cleveland, Ohio; Los Angeles, California; New York City; and Tampa, Florida can use the program to develop healthier habits in their daily lives, prevent disease, and make wise choices in their health care purchases. Members who are considered high-risk or who need chronic care will receive individualized care plans and interventions based on screening, needs, and preference.

By sharpening the focus on health outcomes and access to care, and by tying payments to performance, these new relationships are leading health care reform. The agreement with Aetna, which serves AARP members aged 50 to 64, addresses the needs of an age group for which coverage often is unavailable or unaffordable.

## Conclusion

To transform health care and focus more on health promotion and disease prevention, the private sector must be a key participant. AARP and many other organizations continue to pursue partnership strategies, but we also need presidential leadership on prevention and wellness. A true public-private partnership, congressionally mandated, with enough money and stature behind it to bring everyone to the table, is critical. Educators, corporations (including purveyors of fast and processed food), entities that benefit from and influence our agricultural subsidies, the media, policy makers, insurance, and pharmaceutical groups all need to commit and participate.

Health care reform is potentially one of America's greatest civil rights movements. Prevention and wellness are essential ingredients in this reform. Working together, we can make it happen. Ultimately, it is the only way we will get it done.

## References

[1] Cunningham PJ, Felland LE (2008). Falling behind: Americans' access to medical care deteriorates, 2003-2007, Tracking Report No. 19.

[2] Novelli B (2006). Fifty Plus: Give Meaning and Purpose to the Best Time of Your Life.

[3] (2007). A Purchaser's Guide to Clinical Preventive Services: Moving Science Into Coverage.

[4] Schoen C, Guterman S, Shih A, Lau J, Kasimow S, Gauthier A, Stuyck J, Hollander C (2007). Bending the curve: options for achieving savings and improving value in U.S. health spending.

[5] Franklin D (2008). Just good business: a special report on corporate social responsibility. The Economist.

[6] Morris B (2008). The Pepsi challenge. Fortune.

